# The effect of attending rehabilitation after traumatic knee joint injury on femoral articular cartilage morphology in collegiate rugby players with a history of intracapsular knee joint injury during two-year consecutive rugby seasons

**DOI:** 10.3389/fspor.2023.1309938

**Published:** 2024-01-11

**Authors:** Miyuki Hori, Masafumi Terada, Tadashi Suga, Tadao Isaka

**Affiliations:** ^1^ Faculty of Sport and Health Science, Ritsumeikan University, Kusatsu, Japan; ^2^ Research Organization of Science and Technology, Ritsumeikan University, Kusatsu, Japan

**Keywords:** joint health, ultrasonography, intracapsular injury, osteoarthritis, contact sport

## Abstract

**Introduction:**

This present study aimed to compare ultrasonographic measures of femoral articular cartilage during two-year seasons between collegiate rugby players who have attended supervised rehabilitation following intracapsular knee joint injury and those without a history of knee injury.

**Methods:**

Using a prospective observational study design, 12 male collegiate rugby players with a previous history of intracapsular knee joint injury who have received and completed supervised rehabilitation following their injury and 44 players without knee joint injury participated in this study. Ultrasonographic images were used to verify changes in femoral articular cartilage thickness and cross-sectional area (CSA) with or without a previous history of knee joint injury over two consecutive rugby seasons.

**Results:**

Significant time main effects were observed for the lateral condylar thickness (*p* < 0.001), the intercondylar thickness (*p* = 0.001), the medial condylar thickness (*p* < 0.001), and CSA (*p* < 0.001). No significant interactions nor group main effects were identified for all femoral articular cartilage (*p* < 0.05).

**Conclusions:**

Collegiate rugby players demonstrated a decrease in femoral articular cartilage thickness and CSA over two-year consecutive rugby seasons. These findings indicate that engaging in collegiate rugby induces alterations in femoral articular cartilage structure. Furthermore, there were no differences in all femoral cartilage outcome measures between rugby players with and without a previous history of traumatic knee joint injury. Therefore, attending supervised rehabilitation at the time of their knee joint injury appeared to reduce the impact of a previous history of intracapsular knee joint injury on the change in femoral articular cartilage thickness and CSA among active rugby players.

## 1 Introduction

Participating in sports helps maintain cartilage health but increases the likelihood of sustaining joint injury. In the physically active population, the knee ranks among the most commonly injured joints (1, 2). Injuries to the knee joint alter joint structures and negatively affect the joint's capability to attenuate loading. As a result, the changes in articular cartilage macrostructure and cartilage composition contribute to the early onset of osteoarthritis (OA). Although OA is a common condition among the elderly, OA associated with joint injuries is prevalent among the young populations (2). The term for OA caused by joint injuries is posttraumatic osteoarthritis (PTOA) (1). People who suffer from knee joint injury are four to six times more likely to develop PTOA than those who have no previous history of knee joint injury (1, 3–5). Therefore, knee joint injury becomes a significant risk factor for developing PTOA among the young populations.

Intracapsular knee joint injuries are also the most common (6–8) and severe type of joint injury among rugby players (7, 9). Rugby has a high prevalence of developing symptomatic radiographic knee OA related to a traumatic knee joint injury (10). In a previous study focusing on former rugby players, approximately 40% of the participants experienced a practice reduction of 4 or more weeks due to their injuries (10). Additionally, 39% of all participants had been diagnosed with knee osteoarthritis (OA), and 12% had undergone knee replacement (10). Symptoms of PTOA, such as joint pain (11, 12) and functional limitations (12), interfere with their performances and daily activities (11) and lead to reduced quality of life (11, 12).

Articular cartilage has a resilience that demonstrates a change in its structure when subjected to mechanical stress on the joint and recovers to its baseline (13, 14). A previous study has observed the acute response of femoral articular cartilage thickness following physical activities (15). This ultrasonography (US) study demonstrated femoral articular cartilage thickness changes in response to the mechanical load during walking and drop-landing, and its thickness gradually returned to its baseline thickness over time after ceasing physical activities (15). Several studies observed longitudinal changes in femoral cartilage structures for collegiate athletes (16, 17). A magnetic resonance image (MRI) study has observed compositional and structural changes in the femoral articular cartilage during a competitive season and an off-season for collegiate basketball players (16). This study used quantitative MRI to compare compositional and structural changes in knee articular cartilage between the first pre-season and the second pre-season. Quantitative MRI is an effective way to detect early cartilage degeneration. However, this study did not target populations with a previous history of knee joint injuries (16). In our previous study, we found that femoral cartilage thickness and cross-sectional area (CSA) at the post-season US assessment time point were greater than the pre-season US measures, irrespective of whether participants had a previous history of knee joint injury (17). Additionally, rugby players with a previous knee joint injury history exhibited greater femoral articular thickness and CSA compared to those without such a history (17). An early stage of OA progression may be associated with an increase in articular cartilage thickness (18, 19). The increase in articular cartilage thickness may be attributed to lower US image quality, which could potentially manifest as the blurring of the cartilage margin in the US (20). However, it is insufficient to examine morphological changes in the articular cartilage occurring within a single season. By clarifying the changes in femoral cartilage structure at multi-season, it is necessary to understand the effects of participating in competitions on femoral articular cartilage and the changes in femoral articular cartilage that occur after competitions.

Attending supervised rehabilitation following a traumatic knee joint injury is important to prevent the development of PTOA. Supervised rehabilitation after a knee injury has been shown to restore sensorimotor function and functional joint stability (21–23), potentially mitigating the risk of PTOA. Furthermore, a previous investigation has demonstrated supervised rehabilitation improved articular cartilage composition in patients with a traumatic intracapsular knee joint injury (23). These findings from previous studies indicate that supervised rehabilitation may be beneficial to articular cartilage health in the secondary and tertiary prevention of PTOA after a traumatic intracapsular knee joint injury (21–23). However, it is unclear how receiving and successfully completing rehabilitation at the time of a traumatic knee joint injury affects the changes in femoral articular cartilage at multi-season. This could be useful information for maintaining femoral cartilage health in athletes who continue participating in sports after injury. Therefore, we aimed to evaluate femoral articular cartilage thickness over two consecutive seasons in collegiate rugby players with a history of intracapsular knee joint injuries that had received supervised rehabilitation after their knee injury and those without a history of knee joint injury. We hypothesized that collegiate rugby players with a previous intracapsular knee joint injury, who had received supervised rehabilitation after their traumatic knee injury, would demonstrate no remarkable change in femoral articular cartilage thickness and CSA following two competitive rugby seasons compared with the control players. Furthermore, the thickness of femoral articular cartilage has recovered to the original state at the beginning of the second season compared to the end of the first season.

## 2 Methods

### 2.1 Study design

**Figure 1:**
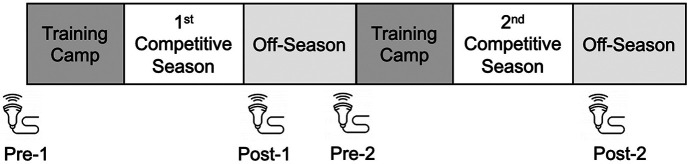
Data collection timeline: Pre-season assessments were performed prior to summer training camp, and post-season assessments were conducted immediately after the conclusion of the seasons.

**Figure 2:**
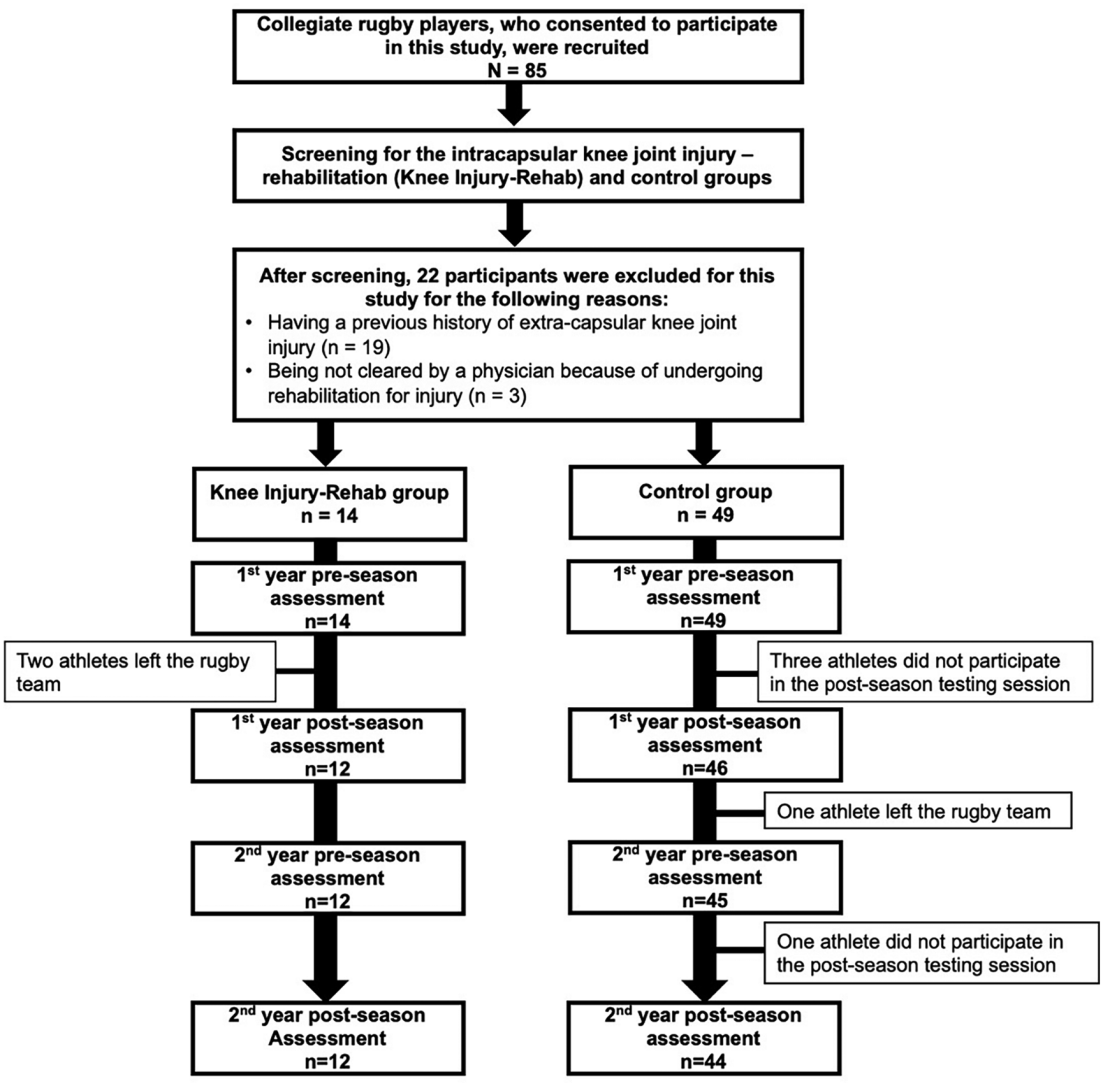
A flow chart for study participants.

This prospective observational study was approved by the Ethics Review Committee for Medical and Health Research Involving Human Subjects at Ritsumeikan University. Before participating in the study, all participants provided informed written consent, and parental or guardian consent was obtained if necessary. This study assessed US outcomes measures of distal femoral cartilage morphology before and after two consecutive rugby seasons in collegiate rugby players with or without a previous history of knee joint injury (Figures 1, 2).

### 2.2 Participants

Eighty-five male rugby players from a Division I collegiate team were recruited for this study. After examining each player's qualifications, 63 players were included in either the injury-rehabilitation history (*n* = 14) or the non-injury history group (*n* = 49). The knee joint injury-rehabilitation history (Injury-Rehab) group was required to (1) have at least one significant previous history of traumatic intracapsular knee joint injuries (e.g., anterior and posterior cruciate ligament sprain, lateral and medial meniscus injury, and femoral cartilage injury) and (2) have received and completed supervised rehabilitation after their knee injury. Because this study focused on the effect of traumatic intracapsular knee joint injury, players with a previous history of extra-capsular knee joint injury (e.g., medial and lateral collateral ligament sprain) were excluded. The control group consisted of rugby players with no previous history of knee joint injury.

During the competitive rugby season, players participated in regular practices and games once a week. Daily practices were held on artificial turf, 1.5–2 h per session, six times a week, and players also had 1–1.5 h of strength training sessions four to five times a week. All participants joined team practices and competitions throughout the seasons. The team's athletic trainer provided all medical records.

### 2.3 Data collection procedures

A single investigator (M.H.) took all femoral cartilage US assessments and analyzed the US examinations. The examinations were performed before (pre-season) and after (post-season) at each competitive season. The pre-season tests were conducted before summer training camp in late July, while the post-season examinations were conducted immediately after the season ended in late December. The following four points of measurement were established in this study: pre-season 1 (Pre-1), post-season 1 (Post-1), pre-season 2 (Pre-2), and post-season 2 (Post-2) (Figure 1).

### 2.4 Ultrasonographic image acquisition

We asked participants not to exercise the day before taking the US image. Upon arriving at the examination room, participants rested in a long-sit position with both knees in full extension for 30 min to unload the cartilage (15). After 30 min of rest, participants laid supine comfortably on an examination table with both knees positioned at 140 degrees of flexion, using a handheld goniometer (17, 24). An ultrasound unit (LOGIQe V2; General Electric Co., Fairfield, CT, USA) with a 12-MHz linear probe was used to obtain US images of the anterior femoral cartilage for this study. The system settings of the portable ultrasound unit were consistent between pre-season and post-season testing sessions and among participants. The linear probe was placed on the distal femoral cartilage of the medial and lateral femoral condyles and above the superior margin of the patella. The US images were oriented to obtain the maximum reflection of the femoral trochlea and the overlying hyaline cartilage, as previously reported (15, 17, 25, 26). The location at which the intercondylar notch was centered on the screen. Three images were recorded at each testing session, with the linear probe being removed and repositioned on the knee between recorded images.

The single investigator (M.H.) assessed all distal femoral cartilage image processing using ImageJ software (National Institutes of Health, Bethesda, MD, USA). The medial and lateral cartilage thickness was estimated at the intercondylar notch and 1 cm apart in the medial and lateral directions (27). The femoral cartilage thickness was measured as the straight-line distance (mm) drawn from the hyperechoic cartilage-bone interface to the synovial space-cartilage interface (17, 26). The CSA (mm^2^) of femoral cartilage was assessed by tracing an area of femoral cartilage between lateral and medial measurement points where femoral cartilage thickness was measured. The US examination of the distal femoral cartilage variables has been demonstrated to be valid (25, 28) and reliable (ICC = 0.83–0.99) (26). Furthermore, before data collection for the current study, the US examiner established good to excellent intratester reliability (ICC_1,3 _= 0.82–0.96).

### 2.5 Statistical analysis

Anthropometric variables were compared between the Injury-Rehab and control groups using independent *t*-tests (*p* < 0.05). For the Injury-Rehab group, the injured limb was used to assess the articular cartilage structures. The control group used the dominant limb, the side of kicking the ball (29). A separated 2 × 4 (group × time) repeated measure ANOVA was used to analyze each US femoral cartilage variable. In the case of the statistically significant interactions and main effects, a Bonferroni *post hoc* univariate analysis with a pairwise comparison was conducted to ascertain the location of significant differences. Statistical significance was set at *p* < 0.05 using SPSS 27.0 (SPSS, Inc. Chicago, IL, USA). Cohen's *d* effect sizes using the pooled standard deviations were calculated to assess the magnitude of differences in each femoral cartilage measurement between independent variables, along with 95% confidence intervals for each pairwise comparison. The strength of effect sizes was interpreted as small (*d* < 0.40), medium (0.40 ≤ *d* < 0.80), or large (*d* ≥ 0.80) (30).

## 3 Results

Fifty-six out of 63 participants (89%) completed all four-time points of femoral cartilage US assessments (Injury-Rehab group = 12 participants, control group = 44 participants) (Figure 2). The type and frequency of intracapsular knee joint injury sustained by the participants were reported in Table 1. Rehabilitation was overseen by orthopedic surgeons, physical therapists, athletic trainers, or a team specializing in conditioning and strength. The supervised rehabilitation lasted for an average of 152 days (minimum: 30 days, maximum: 323 days). The rehabilitation regimens encompassed exercises to restore range of motion, build strength, engage in functional training (balance, proprioception), weight training, plyometrics, aerobic exercises (walking, cycling, running, sprinting), agility, and sport-specific training (tackling, scrimmage, kicking, etc.). The frequency of rehabilitation sessions was tailored to the individual athlete's status and progression. Anthropometric characteristics were not statistically different between the Injury-Rehab and the control groups (*p* < 0.05, Table 2).

**Table 1:** List of a previous history of a traumatic knee joint injury (*n* = 12).

Injury type	Frequency	The number of surgical management
Anterior cruciate ligament (ACL) sprain	4	3
Posterior cruciate ligament (PCL) sprain	6	0
Medial meniscus injury (MMI)	2	1
Lateral meniscus injury (LMI)	2	2
Femoral cartilage injury	1	1

The number of participants who sustained multiple knee joint injury: ACL + LMI = 1, MMI + LMI = 1.

**Table 2:** Demographic characteristics for the intracapsular knee injury-rehabilitation and control groups (*n* = 56).

	Knee injury rehabilitation	Control	*p*-value
	Mean (SD)	Mean (SD)	
*N*	12	44	
Age (years)	19.33 (1.15)	19.43 (0.82)	0.74
Height (cm)	173.17 (6.06)	174.48 (6.67)	0.54
Body mass (kg)	84.33 (12.44)	84.29 (11.43)	0.99
Body mass index (kg/m**^2^**)	28.10 (3.67)	27.66 (3.29)	0.69
# of previous intracapsular knee joint injuries	1.33 (0.49)	0.00	–
	Min = 1, Max = 2		
# of knee joint surgeries	1.58 (0.52)	–	–
	Min = 1, Max = 2		
Time since the most recent inter-capsular knee joint injury (month)	16.96 (13.88)	–	–
	Min = 2, Max = 47		

SD, standard deviation.

**Figure 3:**
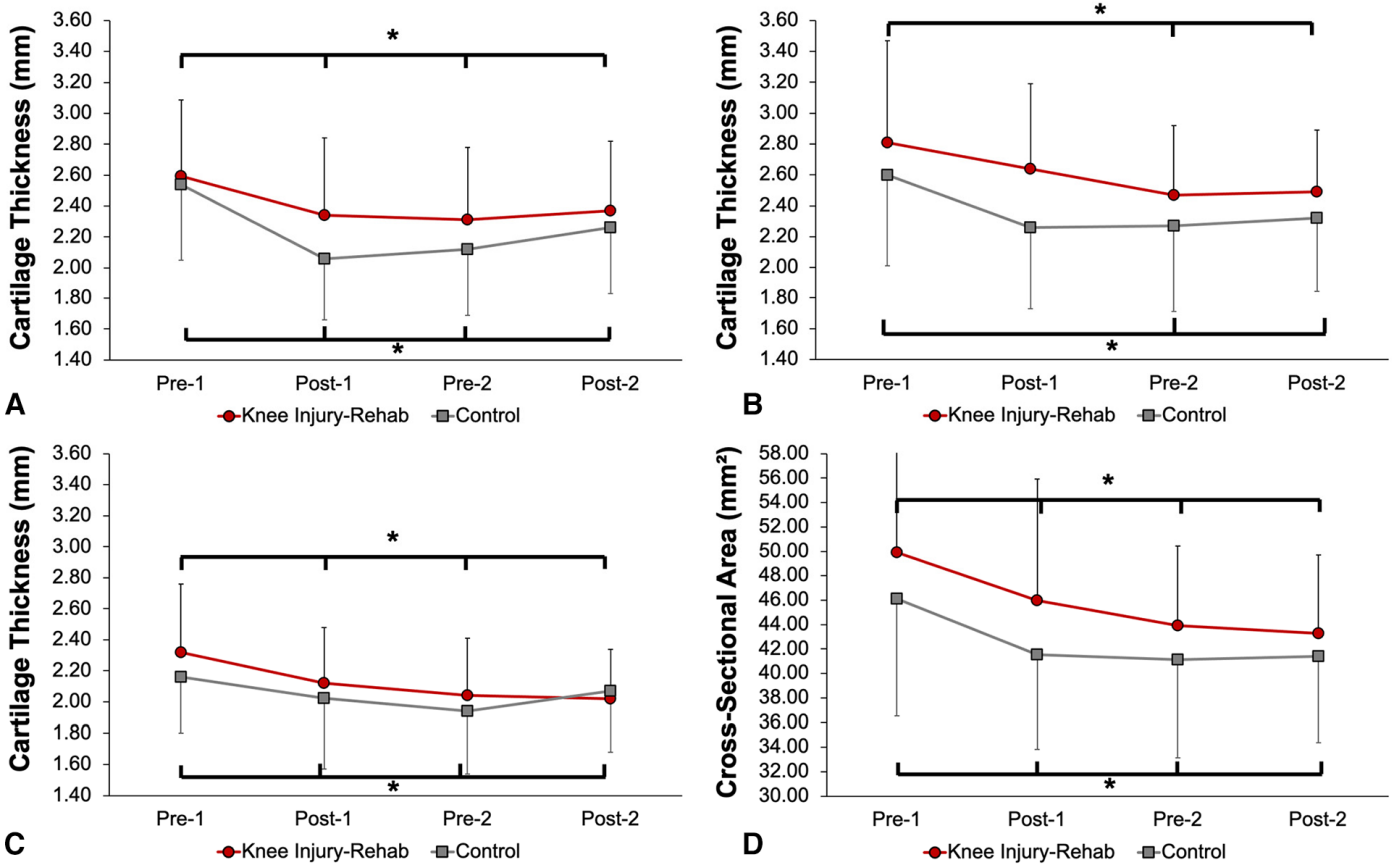
Changes in femoral cartilage variables over two-competitive seasons: (**A**) lateral condylar cartilage thickness, (**B**) intercondylar cartilage thickness, (**C**) medial condylar cartilage thickness, (**D**) cross-sectional area. *Significant time main effects were observed (*p* < 0.05). The femoral articular cartilage variables at the pre-season assessment in the first year (Pre-1) were significantly higher compared to the post-season assessment at the conclusion of the first-year season, the pre-season assessment in the second year (Pre-2), and the post-season assessment in the second year (Post-2) (*p* < 0.05).

The group mean and standard deviation of femoral cartilage variables at the four assessment time points are shown in Figure 3 and Table 3. There were no significant Injury-Rehab main effects for the lateral condylar thickness (*p* = 0.17), the intercondylar thickness (*p* = 1.10), the medial condylar thickness (*p* = 0.51) and CSA (*p* = 0.15). There were no significant Time × Injury-Rehab interactions for the lateral condylar thickness (*p* = 0.47), the intercondylar thickness (*p* = 0.55), the medial condylar thickness (*p* = 0.25), and CSA (*p* = 0.67). Significant Time main effects were observed for the lateral condylar thickness (*p* < 0.001), the intercondylar thickness (*p* = 0.001), the medial condylar thickness (*p* < 0.001), and CSA (p*p* < 0.001) (Tables 3, 4). Compared to Pre-1, there were significantly less lateral condylar cartilage thickness at Post-1, Pre-2, and Post-2 with medium to large effect sizes; intercondylar cartilage thickness at Pre-2 and Post-2 with medium effect sizes; medial condylar cartilage thickness at Post-1, Pre-2, and Post-2 with small to medium effect sizes; as well as CSA at Post-, Pre-2, and Post-2 with medium effect sizes (*p* < 0.05) (Table 4).

**Table 3:** Mean femoral cartilage outcomes: pre- and post-season measurements for two-consecutive years.

Outcomes	Time	Injury-rehab	Control	Main effect/Interaction	*p*-value	*η* ^2^	*β*
		Mean (SD)	Mean (SD)				
Lateral condylar thickness (mm)	Pre-1	2.60 (0.49)	2.54 (0.49)	Time	<0.001*	0.16	1.00
	Post-1	2.34 (0.50)^#^	2.06 (0.40)^#^	Knee injury	0.17	0.04	0.28
	Pre-2	2.31 (0.47)^#^	2.12 (0.43)^#^	Time × knee injury	0.47	0.02	0.21
	Post-2	2.37 (0.45)^#^	2.26 (0.43)^#^				
Intercondylar thickness (mm)	Pre-1	2.81 (0.66)	2.60 (0.59)	Time	0.001*	0.11	0.95
	Post-1	2.64 (0.55)	2.26 (0.53)	Knee injury	1.10	0.05	0.38
	Pre-2	2.47 (0.45)^#^	2.27 (0.56)^#^	Time × knee injury	0.55	0.01	0.17
	Post-2	2.49 (0.40)^#^	2.32 (0.48)^#^				
Medial condylar thickness (mm)	Pre-1	2.32 (0.44)	2.16 (0.36)	Time	<0.001*	0.12	0.98
	Post-1	2.12 (0.36)^#^	2.02 (0.45)^#^	Knee injury	0.51	0.01	0.10
	Pre-2	2.04 (0.37)^#^	1.94 (0.40)^#^	Time × knee injury	0.25	0.03	0.34
	Post-2	2.02 (0.32)^#^	2.07 (0.39)^#^				
Cross-sectional area (mm^2^)	Pre-1	49.91 (9.87)	46.12 (9.57)	Time	<0.001*	0.15	1.00
	Post-1	45.98 (9.96)^#^	41.53 (7.71)^#^	Knee injury	0.15	0.04	0.31
	Pre-2	43.92 (6.51)^#^	41.13 (7.99)^#^	Time × knee injury	0.67	0.01	0.13
	Post-2	43.27 (6.43)^#^	41.38 (6.98)^#^				

SD, standard deviation.

* Significant time main effects were observed (*p* < 0.05).

^#^ The femoral articular cartilage variables were significantly lower compared to these variables at the pre-season assessment in the first year (Pre-1) regardless of group membership (*p* < 0.05).

**Table 4:** Pairwise comparisons between ultrasonography assessment time points and between the injury-rehab and control groups.

Outcomes	Time		*p*-value	Effect sizes (d)	95% CIs		Between-group comparison		*p*-value	Effect sizes (d)	95% CIs	
					Lower	Upper					Lower	Upper
Lateral condylar thickness	Pre-1 vs.	Post-1	0.001*	0.93	0.54	1.32	Injury-Rehab vs. Control	Pre-1	0.74	0.12	−0.52	0.76
		Pre-2	<0.001*	0.84	0.45	1.22		Post-1	0.05	0.66	0.01	1.31
		Post-2	0.03*	0.58	0.20	0.95		Pre-2	0.19	0.43	−0.21	1.08
	Post-1 vs.	Pre-2	1.00	−0.09	−0.46	0.28		Post-2	0.41	0.25	−0.39	0.89
	Pre-2 vs.	Post-2	0.47	−0.27	−0.64	0.10						
Intercondylar thickness	Pre-1 vs.	Post-1	0.07	0.54	0.16	0.92	Injury-Rehab vs. Control	Pre-1	0.30	0.35	−0.29	0.99
		Pre-2	0.01*	0.60	0.22	0.97		Post-1	0.03	0.71	0.06	1.36
		Post-2	0.02*	0.54	0.16	0.92		Pre-2	0.26	0.37	−0.27	1.01
	Post-1 vs.	Pre-2	1.00	0.06	−0.32	0.43		Post-2	0.27	0.37	−0.28	1.01
	Pre-2 vs.	Post-2	1.00	−0.10	−0.47	0.27						
Medial condylar thickness	Pre-1 vs.	Post-1	0.03*	0.36	−0.02	0.73	Injury-Rehab vs. Control	Pre-1	0.21	0.42	−0.22	1.07
		Pre-2	<0.001*	0.60	0.21	0.97		Post-1	0.50	0.23	−0.41	0.87
		Post-2	0.01*	0.34	−0.03	0.71		Pre-2	0.26	0.25	−0.39	0.89
	Post-1 vs.	Pre-2	0.85	0.20	−0.17	0.57		Post-2	0.24	−0.13	−0.77	0.51
	Pre-2 vs.	Post-2	1.00	−0.26	−0.63	0.11						
Cross-sectional area	Pre-1 vs.	Post-1	0.01*	0.49	0.12	0.87	Injury-Rehab vs. Control	Pre-1	0.23	0.39	−0.25	1.04
		Pre-2	0.01*	0.59	0.20	0.97		Post-1	0.10	0.54	−0.10	1.19
		Post-2	0.01*	0.61	0.24	0.99		Pre-2	0.65	0.36	−0.28	1.00
	Post-1 vs.	Pre-2	1.00	0.09	−0.28	0.47		Post-2	0.40	0.28	−0.37	0.92
	Pre-2 vs.	Post-2	1.00	−0.01	−0.38	0.36						

CI, confidence interval.

* The femoral articular cartilage variables were significantly lower compared to these variables at the pre-season assessment in the first year (Pre-1) regardless of group membership (*p* < 0.05).

## 4 Discussion

This study aimed to examine structural changes in the femoral cartilage in collegiate rugby players with a previous history of intracapsular knee joint injury who have received and successfully completed supervised rehabilitation after their previous injury and those without a history of knee joint injury for two competitive rugby seasons. The findings of this study were that rugby players demonstrated a decrease in femoral articular cartilage thickness and CSA from Pre-1 to Post-2, supported by medium to large effect sizes with 95% confidence intervals that did not cross zero. These findings indicate that engaging in collegiate rugby induces alterations in femoral articular cartilage structure, and the reduction in cartilage thickness and CSA were clinically relevant. It prompts consideration of necessary measures to preserve athletes' cartilage health and mitigate the risk of knee OA. Additionally, we found no differences in lateral condylar thickness, the intercondylar thickness, the medial condylar thickness, and CSA between those with and without a previous history of traumatic knee joint injury. These results indicate attending supervised rehabilitation after a knee injury may reduce impacts of a previous history of intracapsular knee joint injury on the changes in femoral articular cartilage thickness among collegiate rugby players. However, it is important to acknowledge that these findings are limited to the short term, and we cannot extend the implications of attending supervised rehabilitation on cartilage health beyond the study duration.

In this study, we observed a decrease in femoral cartilage thickness among participants during competitive rugby seasons. There were medium and large effect sizes for lateral condylar and intercondylar cartilage thickness and CSA at Post-1, Pre-2, and Post-2 compared to Pre-1, and 95% confidence intervals around the effect sizes did not cross zero. The effect size for medial condylar thickness between Pre-1 and Pre-2 was medium with 95% confidence intervals that did not overlap zero. The medium and large effect sizes with narrow 95% confidence intervals bolstered our results by highlighting the moderate to large magnitude of reduction in articular cartilage thickness and CSA during two consecutive rugby seasons. It has been assumed that differences in loading environment may have a significant influence on the morphological characteristics of articular cartilage (31). During rugby matches, players engage in a spectrum of activities, including low- and high-intensity running, rapid accelerations and decelerations, and high-intensity contact and set plays (31, 32). The increased repetitive shear loading from sudden lateral movements, braking, and acceleration in rugby may reduce femoral articular cartilage thickness and the fatigue life of cartilage (18), potentially contributing to an elevated risk of knee OA (33). Femoral articular cartilage thickness seems to be load-dependent (34, 35). Previous studies observed that exercises, such as walking, running, and drop-landing produced greater femoral cartilage deformation compared to a non-exercise condition (15, 26). However, it is unclear how different movement and joint loading patterns would be in terms of changes in cartilage thickness. Therefore, future investigation is required to examine morphological responses of femoral articular cartilage to different movement conditions, such as sudden lateral movements.

The observed decrease in femoral cartilage thickness over two-year competitive seasons in this study suggests a potential alteration in the distribution of mechanical stresses within the knee joint. Changes in femoral cartilage thickness are often considered indicative of alterations in its ability to withstand mechanical stress (24). A reduction in articular cartilage thickness may lead to a change in the loading area on the knee joint, suggesting it may not be fixed to a specific point (36). Additionally, a previous study demonstrated that this reduction in articular cartilage thickness led to a decrease in the contact area between the cartilage and meniscus (37), impacting the meniscus's capacity to efficiently transmit load. Thus, the findings may explain why participation in rugby is linked to a heightened risk of OA. Further research is needed to inform our understanding of types of loading for rugby players to reduce the risk for knee OA and to design prevention programs that facilitate cartilage health.

Changes in biomechanics due to injuries can potentially lead to modifications in contact mechanics, possibly increasing stress within the knee joint (36). Previous studies have reported an increase in femoral articular cartilage thickness after six months (38), one year (39), three years (40), four years (36), and five years after anterior cruciate ligament (ACL) reconstruction (24). However, the current study did not observe significant differences in any femoral cartilage thickness variables between rugby players with and without a previous history of knee joint injury. This study encompasses all intraarticular knee joint injuries and not just an ACL injury. It is possible that different changes in cartilage thickness, distinct from ACL injuries, may have influenced the absence of significant differences in the morphological changes of the femoral cartilage. For future research, exploring cartilage degeneration in not only intracapsular knee joint injuries but also various other knee-related conditions, such as extra-capsular knee joint injury and muscle-tendon strain, would be advantageous. This approach could contribute to maintaining a significant role in preserving the cartilage health in athletic populations.

Participants in this study, who sustained a knee joint injury, were undergoing rehabilitation programs by a team physician and a team athletic trainer to return to play at the competitive level. The rehabilitation program included various components to restore knee joint function, such as strengthening, range of motion exercises, and neuromuscular training (40, 41). Several studies have reported that an increase in knee extensor muscle strength effectively reduces pain (42, 43) and improves physical function (43). By continuing training after returning to rugby, participants can enhance muscle strength and functional performance, potentially delaying the early onset of PTOA (12). It is speculated that such rehabilitation programs and training may have prevented significant changes in femoral cartilage structures, even in individuals with a previous history, thus explaining the absence of observed alterations. However, we did not compare US measures of femoral articular cartilage morphology between rugby players who did not attend supervised rehabilitation at the time of their knee joint injury and those who did. Thus, future research will be essential to elucidate the long-term impact of supervised rehabilitation after a traumatic knee joint injury.

While no significant Injury-Rehab main effects were observed for the selected cartilage variables, some of the non-significant between-group difference in cartilage thickness were associated with medium effect sizes, with 95% confidence intervals that did not cross zero. Specifically, the Injury-Rehab group exhibited greater lateral condylar and intercondylar cartilage thickness at Post-1 compared to the control group. The medium effect sizes indicate that these differences are likely of moderate magnitude, suggesting clinically meaningful differences between groups. In instances of medium effect sizes with 95% confidence intervals extending across zero, these associations may be prone to statistical error and could benefit from a larger sample size to strengthen the findings. Previous studies reported greater femoral cartilage thickness in individuals with a history of a traumatic intracapsular knee joint injury compared to control participants (17, 44). Researchers have hypothesized that an increase in articular cartilage thickness may be attributed to cartilage swelling and/or hypertrophy of the extracellular matrix (45, 46) as well as be related to a progression of OA (18, 19). If the articular cartilage in the Injury-Rehab group is thicker compared to the control group, it appears that rehabilitation did not confer any benefits to the cartilage. Therefore, developing more effective interventions is crucial to modifying the adverse impacts of knee joint injuries on cartilage health.

This present study also examined the changes in femoral articular cartilage thickness and CSA during an off-season (Post-1 to Pre-2). A previous MRI study observed structural and compositional alterations in femoral articular cartilage during an off-season (16). Additionally, a study investigating acute change in femoral cartilage reported that thinning cartilage due to 30 min of exercise returns to baseline thickness over time after the completion of exercise (15). Based on these studies (15, 16), a hypothesis was proposed that undergoing off-season with reduced exercise load would allow the thinning cartilage during the competitive season to recover to baseline. However, in this study, the cartilage thickness did not recover during the off-season. We observed that the changes in femoral cartilage thickness ranged from −0.08 mm to 0.04 mm during the off-season. Small effect sizes were noted, accompanied by 95% confidence intervals that overlapped zero when comparing US cartilage variables between Post-1 and Pre-2. The presence of small effect sizes, coupled with wide 95% confidence intervals, indicates that the reduction in articular cartilage thickness and CSA during the off-season was of minor magnitude. Notably, these changes in femoral cartilage thickness did not exceed the established minimal detectable change (0.09 mm–0.15 mm) (15, 26).

The previous study targeting collegiate basketball players defined the period without team practice as the off-season (16). On the other hand, we defined the off-season as the period from the end of the competitive season to the beginning of the next competitive season. As described in our study, the off-season encompassed a time when regular team practices were suspended, and athletes engaged in fundamental training and skill development in preparation for the upcoming competitive season. Although not on a regular basis, practice matches were also conducted. The strain exerted on the cartilage during such training sessions might explain the absence of morphological recovery in the cartilage. While articular cartilage possesses resiliency (13), the accumulation of training load on the joint can lead to overload, impeding the generation of essential components required to maintain the cartilage structure (47). A study on collegiate basketball players found altered femoral cartilage composition during the off-season, suggesting that the off-season strength and conditioning training may place more stress on the femoral cartilage (16). However, the authors observed some recovery in the anterior region of the femoral articular cartilage during the off-season (16). This finding implies that incorporating a designated period of rest and recovery, such as the off-season, can aid in restoring the cartilage structure altered by physical activity. In competitive sports, the off-season does not necessarily denote complete inactivity. Therefore, investigating the types of exercises and exercise loads that aid in maintaining and enhancing cartilage health would significantly contribute to extending the athletic careers and longevity of competitive athletes. For example, self-paced running has demonstrated its potential to stimulate improvements in cartilage health without elevating the risk of knee OA (48). It remains a challenge to determine the specific exercises and joint load most beneficial for training during the rest period.

A limitation of this study was that we did not consider participants' self-reported function. Even though participants actively engaged in team practices and games, we acknowledge the possibility that some may have experienced pain and functional limitations in both rugby and daily activities. Prior studies utilized self-reported questionnaires, such as the Knee Osteoarthritis and Injury Outcome Score (KOOS), to assess the impact of knee joint injuries (41, 49). These studies highlighted significantly lower KOOS scores in the knee ligament injury group, particularly in the symptoms and knee-related quality of life subscales (41, 49). Long-term tracking and assessment using knee-related self-reporting questionnaires may shed light on structural changes in the degenerated knee joint of individuals with a history of knee joint injuries.

In this study, a single examiner conducted all tasks, from collecting US data to assessing femoral articular cartilage. All data were anonymized to prevent individual identification, and maintaining blinding throughout the study was another limitation. Future studies should encompass measurements and investigations across multiple teams at the same competition level to facilitate a more comprehensive analysis.

## 5 Conclusion

We observed a significant decrease in femoral articular cartilage thickness and CSA during two consecutive rugby seasons in a collegiate rugby population. Participating in a competitive rugby season appeared to significantly impact the change in femoral articular cartilage thickness and CSA among active rugby players. Furthermore, we did not find an association between changes in femoral articular cartilage morphology and a previous history of intracapsular knee joint injury in collegiate rugby players. Attending supervised rehabilitation at the time of their knee joint injury may have a positive effect on femoral articular cartilage health in collegiate rugby players with a previous history of intracapsular knee joint injury. Future research should continue to monitor multi-season changes in femoral articular cartilage thickness, focusing on both knee joint injury status and participation in rugby competitions.

## Data Availability

The raw data supporting the conclusions of this article will be made available by the authors, without undue reservation.
